# Neonatal Porcine Germ Cells Dedifferentiate and Display Osteogenic and Pluripotency Properties

**DOI:** 10.3390/cells10112816

**Published:** 2021-10-20

**Authors:** Mohammad Amin Fayaz, Gustavo dos Santos Rosa, Ali Honaramooz

**Affiliations:** Department of Veterinary Biomedical Sciences, Western College of Veterinary Medicine, University of Saskatchewan, Saskatoon, SK S7N 5B4, Canada; ma.fayaz@usask.ca (M.A.F.); gustavo.s.rosa@unesp.br (G.d.S.R.)

**Keywords:** gonocytes, testis cells, culture, trans-differentiation, pluripotency

## Abstract

Gonocytes are progenitors of spermatogonial stem cells in the neonatal testis. We have previously shown that upon culturing, neonatal porcine gonocytes and their colonies express germ cell and pluripotency markers. The objectives of present study were to investigate in vitro trans-differentiation potential of porcine gonocytes and their colonies into cells from three germinal layers, and to assess pluripotency of cultured gonocytes/colonies in vivo. For osteogenic and tri-lineage differentiation, cells were incubated in regular culture media for 14 and 28 days, respectively. Cells were cultured for an additional 14 days for osteogenic differentiation or 7 days for differentiation into derivates of the three germinal layers. Osteogenic differentiation of cells and colonies was verified by Alizarin Red S staining and tri-lineage differentiation was confirmed using immunofluorescence and gene expression analyses. Furthermore, upon implantation into recipient mice, the cultured cells/colonies developed teratomas expressing markers of all three germinal layers. Successful osteogenic differentiation from porcine germ cells has important implications for bone regeneration and matrix formation studies. Hence, gonocytes emerge as a promising source of adult pluripotent stem cells due to the ability to differentiate into all germinal layers without typical biosafety risks associated with viral vectors or ethical implications.

## 1. Introduction

Gonocytes are a transitory population of male germline stem cells (MGSCs) in the neonatal testis; they develop from fetal primordial germ cells (PGCs) and postnatally transition into spermatogonial stem cells (SSCs). In mice, gonocytes proliferate within the testicular niche by embryonic day (ED) ~12.5, gradually become mitotically inactive by ED ~15.5, and then maintain their quiescent state for the remainder of the prenatal period [1,2]. The transition of gonocytes into SSCs occurs at ~6 days after birth in rodents or ~2 months in humans [3]. SSCs, considered adult stem cells, are fundamental for spermatogenesis due to their dual potential for self-renewal and differentiation ability for subsequent mitosis and meiosis [4,5]. Compared to both PGCs and SSCs, gonocytes in the neonatal testis are a preferred source of MGSCs, since they offer a relatively large population, ease of accessibility, identifiable morphology, and more specific molecular markers for their identification and isolation [6].

When still situated in the testis niche, MGSCs are programmed to unipotently differentiate into more specialized germ cells [7,8]. However, compiling evidence demonstrates the dedifferentiation potential of MGSCs, as well as their ability to derive multipotent or even pluripotent stem cells in an ex situ environment [9,10,11,12,13,14]. These cells can spontaneously transform into a population of cells that express embryonic stem (ES) cell markers, otherwise if genetic manipulations were needed, they would have imposed safety concerns regarding their use and expansion [9,15,16,17]. The ability to dedifferentiate into a pluripotent state allows these ES-like cells to convert into other cell types from derivatives of three germinal layers [9,10,11,13].

Although several studies have demonstrated the potential of MGSCs as a source of pluripotent stem cells, the extent of their full potential is yet to be known and the complicated derivation process remains a major limitation in their application. Further, the conversion of MGSCs into a dedifferentiated state in the presence of exogenous factors, such as multiple growth factors and embryonic fibroblasts used as the supportive feeder layer, may pose unwanted genetic and epigenetic changes to the dedifferentiated cells; this, too, may limit their applications. Additionally, passaging of MGSCs for their expansion or induction of reprogramming can disrupt their normal cell cycle, growth, and overall physiological response to their microenvironment [9]. Therefore, the use of minimal exogenous factors and manipulations for induction of pluripotency in prospective studies is desirable for reducing concerns of immunogenicity, tumorigenicity, and/or viral contamination, and provides a more practical approach for in vitro development [8,13].

Pluripotent stem cells have also been produced by introducing specific sets of pluripotency-associated genes or ‘reprogramming factors’ to the somatic cells. The resultant cells, known as induced pluripotent stem (iPS) cells, were developed via retroviral delivery of various transcription factors (e.g., Oct3/4, Sox2, c-Myc, and Klf4) to adult dermal fibroblasts [18,19,20], hepatocytes, gastric epithelial cells, and even neural stem cells [21,22]. However, the practicality of utilizing different stem cells in clinical settings remains limited due to the ethical and safety concerns over their source [23]. In addition, current protocols for inducing differentiation into specific cell lineages are inefficient, time-consuming, technically complex, and are not cost effective given the requirement for multiple induction materials [24,25]. Increased tumorigenicity of iPS cells due to introduced transcription factors, genetic instability, and biosafety risks associated with the use of viral vectors in their development further limits their applications as therapeutic agents [13]. As a result, it is imperative to seek alternative stem cell sources that lack the biosafety risks associated with viral transduction or tumor formation, and the ethical concerns associated with the manipulation of embryos to harvest stem cells. 

Our laboratory has recently shown that porcine gonocytes, cocultured with testis somatic cells as a feeder layer, rapidly expand in our developed culture conditions composed of basic media and fetal bovine serum (FBS), with or without basic fibroblast growth factor (bFGF). More importantly, we demonstrated that porcine gonocytes are capable of spontaneous dedifferentiation into more primitive stages that express ES cell markers such as SSEA-1, POU5F1, NANOG, and E-cadherin [17]. This is especially important since in vitro dedifferentiation and trans-differentiation of germ cells in previous reports required coculturing with somatic cells from other species and/or supplementation of the media with multiple exogenous factors [7,13,23]. In the present study, we used our recently developed culture conditions to obtain a population of ES-like cells that express pluripotency markers from porcine gonocytes, and then further evaluated their teratoma formation potential using an in vivo implantation assay. We also investigated the induction of their differentiation into derivatives of all three germinal layers by using a simple protocol without the addition of multiple exogenous factors. In doing so, the present study provides a much faster and safer approach for the acquisition of differentiated germinal cells than that reported in previous studies, without complicated manipulation procedures and a reduced overall differentiation cost.

## 2. Materials and Methods

### 2.1. General Experimental Design

**Figure 1:**
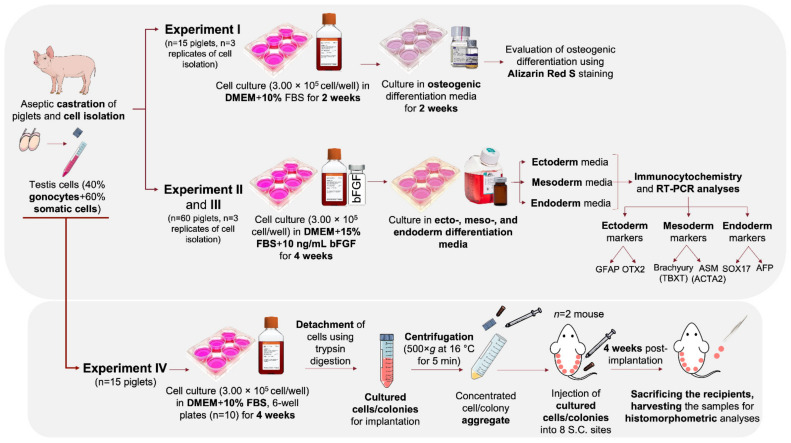
Schematic representation of the experimental design. In Experiment I, differentiation of neonatal porcine gonocytes to osteogenic pathway was investigated. Gonocytes were isolated from neonatal piglets and cultured in regular media for 2 wk to induce dedifferentiation. Media was then changed to osteogenic differentiation media to direct their differentiation into osteogenic cells. Cells and colonies were stained with Alizarin Red S for confirmation of osteogenic differentiation. In Experiment II and III, to induce tri-lineage differentiation, porcine gonocytes were cultured in regular media for 4 wk to induce dedifferentiation. Cells and colonies were then cultured in ectoderm, mesoderm, or endoderm differentiation media to direct their differentiation into the respective cell derivates. Tri-lineage differentiation was confirmed by immunocytochemistry and RT-PCR analyses against markers associated with formation of ectoderm, mesoderm, and endoderm. The pluripotency and tumorigenicity of cultured gonocytes and their colonies were also investigated using an implantation assay (Experiment IV). Isolated testis cells were cultured for 4 wk in DMEM + 10% FBS, dislodged from culture wells, and injected into the subcutaneous tissue of immunodeficient recipient mice. After 4 wk, recipient mice were sacrificed, and implants were retrieved for histomorphometric analyses.

Figure 1 shows the schematic representation of the experimental design. The present study was comprised of four sets of experiments used to investigate the differentiation potential and in vivo teratoma formation of cultured neonatal porcine gonocytes. In Experiment I, cultured gonocytes were directed to differentiate into an osteogenic pathway, where differentiation was confirmed using Alizarin Red S staining. In Experiment II and III, the cultured cells were directed to differentiate into derivatives of the three germinal layers (e.g., ectoderm, mesoderm, and endoderm) followed by confirmation using immunocytochemistry and expression analysis for lineage-specific markers. In Experiment IV, the in vivo teratoma formation of cultured cells was tested using an implantation assay as a follow up to our previous report of their expression of several pluripotency markers [17]. 

### 2.2. Castration of Donors, Testis Cell Isolation, and Culture in Regular Media

Procedures for the castration of neonatal piglets (~1-wk old; *n* = 15 for Experiment I, *n* = 60 for Experiment II and III, and *n* = 15 for Experiment IV), testis cell isolation, and cell culture were conducted as previously described [14,26,27]. Cells were cultured in Dulbecco’s modified Eagle’s media (DMEM; catalogue No. 12-604F; Corning, New York, NY, USA) supplemented with 10% fetal bovine serum (FBS; catalogue No. A15-701; PAA Laboratories, Etobicoke, ON, Canada) for 14 days in Experiment I, 28 days for Experiment IV, and in DMEM + 15% FBS + 10 ng/mL basic fibroblast growth factor (bFGF; catalogue No. 233-FB-025; R&D Systems, Minneapolis, MN, USA) for 28 days in Experiment II. However, in Experiment II, following immunocytochemistry, the cells and colonies did not stain positive for the selected differentiation markers. Also, cells showed a reduced confluency following visual examination on day 4 of differentiation in Experiment II. As such, we designed Experiment III where cultures were exposed to the various differentiation media for a longer period and without frequently changing the media to prevent potential interference with cell organization and interactions. In Experiment III, similar seeding density, regular media composition, and incubation conditions were used as in Experiment II. 

### 2.3. In Vitro Osteogenic Trans-Differentiation of Porcine Germ Cells

The osteogenic differentiation media was prepared according to the manufacturer’s instructions (catalogue No. A1007201; StemPro Osteogenesis Differentiation Kit, Gibco, Grand Island, NY, USA). Briefly, differentiation supplement was mixed with base media at a 1:9 ratio. On day 14 of culture, regular media was removed, and each well was rinsed with Dulbecco’s phosphate-buffered saline (DPBS; catalogue No. 20-031-CV; Mediatech, Manassas, VA, USA) and cells were exposed to the prepared osteogenesis differentiation media for 14 days. This differentiation media was replenished every 3 days and cultures were visually examined using phase-contrast microscopy.

### 2.4. In Vitro Tri-Lineage Differentiation of Porcine Germ Cells

For trans-differentiation of gonocytes and colonies into derivatives of the three germinal layers, tri-lineage differentiation kits were used according to the manufacturer’s instructions with modifications. On day 28 of Experiment II, regular media in different wells was switched with each tri-lineage differentiation media that was developed for differentiation of human pluripotent stem cells (catalogue No. SC031B ectoderm kit; catalogue No. SC030B mesoderm kit; and catalogue No. SC019B endoderm kit; all from R&D Systems). To prepare the endoderm differentiation media-I, bFGF, Activin A, and Wnt-a were diluted 1000-fold in pre-warmed differentiation base media. To prepare the endoderm differentiation media-II, only bFGF and Activin A were diluted 1000-folds in pre-warmed differentiation base media. Each tri-lineage differentiation media was tested in a 6-well plate containing three replicates. For ectoderm and mesoderm differentiation, regular media was switched with 2.5 mL of the differentiation media and cells were incubated for 24 h at 37 °C and 5% CO_2_. The media was replenished with fresh differentiation media every 24 h on days 2 and 3. For endoderm differentiation, regular media was switched with differentiation media-I and plates were maintained in similar conditions as described above. Media-I was then replaced with media-II after 16 h of incubation. Media-II was replenished every 12 h on days 2 and 3. Alternatively, in Experiment III, cultures were incubated with 2.5 mL of the ectoderm or mesoderm differentiation media for 7 days, but without regularly replenishing the media. For endoderm differentiation, regular media was replaced with differentiation media-I, media-I was switched with media-II after 16 h of incubation, and the cultures were maintained for an additional 7 days without replenishing the media. In each differentiation group, cultures were examined daily under a phase-contrast microscope.

### 2.5. Imaging and Morphometrical Assessment

Viability of freshly isolated and cultured cells was evaluated using the trypan blue exclusion technique [14,26,27]. For quantification and measurements in Experiment I, photomicrographs were captured from culture plates in treatment and control groups before and after staining with Alizarin Red S. In Experiment II and III, photomicrographs were obtained before adding the differentiation media (day 0), as well as on days 3, 5, and 7 after incubation with differentiation media. Imaging, morphometrical assessment, and quantification protocols were carried out as described in our previous work [17].

### 2.6. Staining and Immunocytochemistry for Evaluation of Dedifferentiation and Trans-Differentiation

#### 2.6.1. Alizarin Red S Staining

Calcium deposition is a known indicator of successful differentiation of stem cells into osteoblasts, and of in vitro bone matrix formation [28]. Therefore, on day 14 of differentiation in Experiment I, Alizarin Red S staining solution was used to identify calcium deposition as described previously [29]. Briefly, the media was aspirated, wells were rinsed with DPBS, and cells/colonies were fixed using 10% formaldehyde solution (catalogue No. HT501128; Sigma-Aldrich) for 30 min. Wells were rinsed and incubated with the staining solution at room temperature for 20 min. Wells were rinsed again with distilled water and visualized under an inverted light microscope.

#### 2.6.2. Immunofluorescence Assay

In Experiment II and III, cells and colonies were grown on poly-L-lysine-coated coverslips. The preparation of coverslips followed our previously described protocols [26]. We have previously shown that culture of neonatal porcine MGSCs in regular media leads to their dedifferentiation and expression of multiple ES cell markers [14,17]. As such, here we also evaluated the expression of POU5F1 as a representative ES marker to confirm MGSC dedifferentiation prior to their trans-differentiation [30,31,32]. The expression of this marker was examined immediately after cell isolation and on day 28 of culture in regular media. On day 4 of Experiment II and day 7 of differentiation in Experiment III, the coverslips were removed from culture wells and stained against gonocyte- and germinal layer markers, as summarized in Table 1. The procedure used for immunofluorescence assay has been previously described by our laboratory [14,26]. To double-label the cells and colonies with a gonocyte-specific marker, FITC-labeled Dolichos biflorus agglutinin (DBA-FITC; catalogue No. FL1031; Vector Labs, Burlington, ON, Canada) was used [33]. 

### 2.7. Gene Expression Analyses for Confirmation of Dedifferentiation and Trans-Differentiation

In Experiment III, the expression of the pluripotency marker (POU5F1) and 6 germinal layer markers were examined using reverse-transcriptase polymerase chain reaction (RT-PCR). To confirm dedifferentiation of cultured MGSCs, expression of POU5F1 was examined using samples of freshly-isolated testis cells and cultured cells in regular media on days 7, 14, 21, and 28. Following 7 days of culture in differentiation media, RT-PCR analysis for differentiation markers was carried out on cells and colonies from each differentiation group. The procedure for gene expression analyses was as described in our previous work [17]. Selected target genes were as follows: *POU5F1, OTX2*, *GFAP*, *TBXT* (T-box transcription factor T; gene for Brachyury), *ACTA2* (Actin alpha 2; gene for ASM), *SOX17*, and *AFP*. The annealing temperatures, names, and primer sequences for each gene are listed in Table 2. 

### 2.8. Subcutaneous Implantation of Cultured Cells and Colonies

Cultured cells were examined for pluripotency based on their teratogenic potential following subcutaneous injection into male hairless immunodeficient mice (*n* = 2; SHO, Crl: SHO-*Prkdc^scid^Hr^hr^*, strain code 474; Charles River, Montreal, Canada). On the day of implantation all media (DMEM + 10% FBS) was removed, the wells were rinsed with sterile DPBS, and cells/colonies were detached using trypsin-EDTA solution as previously described [26]. To stop the trypsin reaction, FBS was added to each well, then contents were pooled and centrifuged at 500× *g* at 16 °C for 5 min. The supernatant was discarded, and the pellet washed with DPBS and centrifuged again using the same settings. Preparation and anesthesia of recipient mice were as described previously [34,35,36]. For implantation procedure, 0.1 mL of the cell aggregates (equals to the content of ~3 wells) were injected subcutaneously into each of 8 sites per mouse (4 sites on the left and 4 sites on the right side of the dorsal midline) as described previously [34,35,36]. Each mouse was kept in an individual plexiglass micro-isolator cage under sterile conditions and maintained in standard temperature (22 ± 2 °C) and controlled lighting photoperiod (12 h light/dark cycles). The mice were kept for 4 wk, evaluated daily for abnormal signs, and provided with sterile water and chow *ad libitum*.

### 2.9. Retrieval of the Subcutaneous Implants and (Immuno)histochemistry

Recipient mice were sacrificed after 4 wk. The implants were retrieved, rinsed with DPBS, fixed in Bouin’s solution, processed, embedded in paraffin, and sectioned at 5 µm. Among the largest sections, randomly selected samples were used for routine histology (hematoxylin and eosin; H&E staining) and immunohistochemistry against lineage-specific antibodies (i.e., GFAP, ASM, and AFP). The procedure for immunohistochemistry was performed as described previously [35].

### 2.10. Statistical Analyses

All the data are presented as the mean ± standard error of mean (SEM) from at least three independent replicates and *p* < 0.05 was considered as statistically significant. The statistical methods used for the analyses include *t*-test and one-way analysis of variance (ANOVA), and Tukey’s HSD was used as the post hoc test, unless stated otherwise. All data analyses were performed using the Statistical Package for Social Sciences (IBM SPSS Statistics for Macintosh, Version 26.00; IBM Corporation, Armonk, NY, USA).

## 3. Results

### 3.1. Viability and Morphometric Assessments

**Figure 2:**
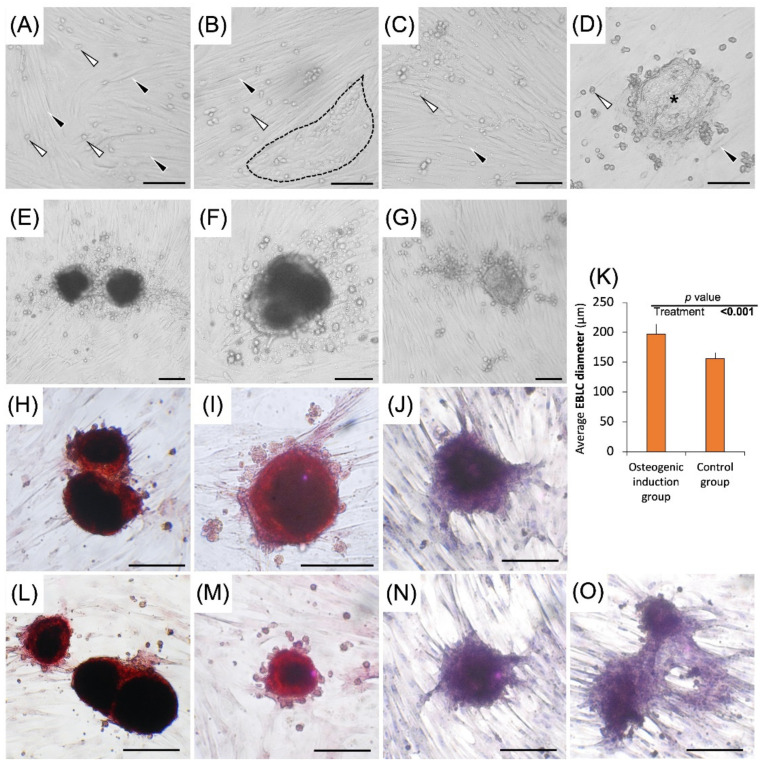
Representative photomicrographs of culture wells in regular media and osteogenic differentiation media, as well as the average diameter of EBLCs in different groups. When cultured in regular media, by day 7, somatic cells of the testis formed a complete monolayer attached to the culture well (black arrowhead). Gonocytes were located on top of the spindle-shaped somatic cells (white arrowhead) (**A**). By day 14, somatic cells formed circular arrangements (black broken line) (**B**). In circular arrangements, gonocytes were surrounded by cytoplasmic projections of somatic cells. Gonocytes gradually increased in number by undergoing proliferation. Also, by day 14, convergence of surrounding gonocytes in circular arrangements led to formation of large multinucleated embryoid body-like colonies (EBLCs). By day 28, gonocytes increased in number and EBLCs became larger (black asterisk) (**C**,**D**). EBLCs in osteogenic differentiation group (**E**,**F**,**H**,**I**,**L**,**M**) possessed a more compact appearance compared to those developed in control groups (**G**,**J**,**N**,**O**). Also, EBLCs in osteogenic differentiation groups developed darker central areas and narrower translucent marginal areas. This may be related to their dome-shaped structure leading to a thicker central area compared to thinner marginal areas. EBLCs appeared positive for Alizarin Red S staining, indicative of their transformation into calcium deposition sites (**H**,**I**,**L**,**M**), while control groups appeared negative for this staining (**J**,**N**,**O**). Although the number of EBLCs did not differ between osteogenic differentiation and control groups, their average diameter was greater in differentiation group (*p* < 0.001) (**K**) (Scale bar = 100 µm).

The viability of the freshly isolated testis cells and cultured cells sampled on day 28 in the osteogenic differentiation group, (Experiment I) and day 35 in the tri-lineage differentiation groups (Experiment III), was ~90%. In Experiments I-III and during the culture period in regular media, morphological features of the cells and colonies were similar to those reported in our previous study, including formation of a wave-shape monolayer after 7 days, development of circular arrangements, and 3D embryoid body-like colonies (EBLCs) after ~14 days [17] (Figure 2A–D).

In Experiment I, EBLCs in the control group appeared light in color, while those developed in the differentiation induction media had a more compact appearance with a darker center. The margins of EBLCs developed in differentiation induction media were relatively translucent compared to their opaquer central areas (Figure 2E–J). The number of EBLCs did not differ between these groups (*p* = 0.56), although the average EBLC diameter was ~26% greater in the differentiation induction media compared to control groups (*p* < 0.001; Figure 2K). The average EBLC diameter in the differentiation and regular cultures were 197.16 ± 16.66 µm and 156.10 ± 10 µm, respectively.

In Experiment II, cell confluency was reduced following incubation with tri-lineage differentiation media in all treatment groups. Hence, Experiment II was removed from further analyses. Following this, Experiment III was designed and conducted, wherein a modified media changing regimen was used to reduce cell loss and interference with cell organization and arrangement.

**Figure 3:**
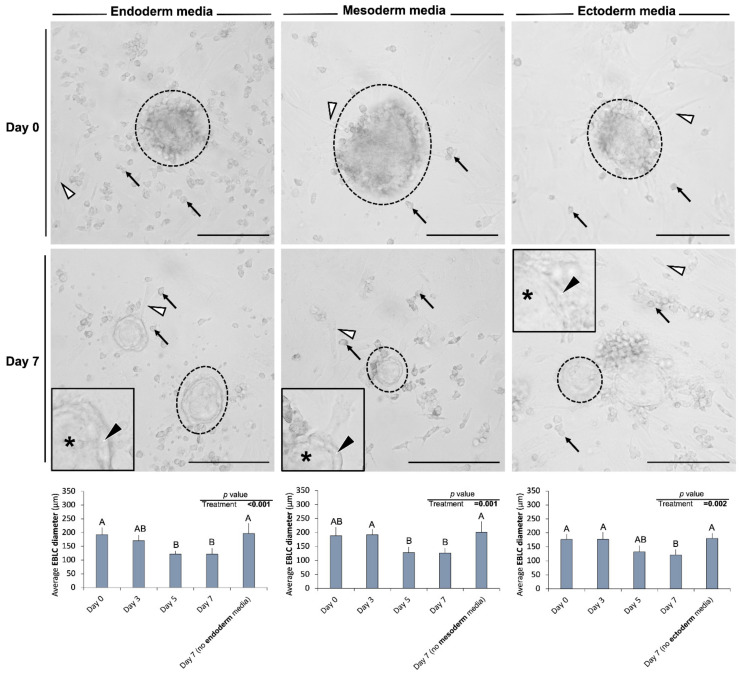
Representative photomicrographs obtained from culture wells before (day 0) and 7 days after incubation with the tri-lineage differentiation media. The overall morphological features of gonocytes (black arrows), embryoid body-like colonies (EBLCs) (dotted circles), and somatic cell monolayer (white arrowheads) did not differ between control groups provided with regular media (DMEM + 15% FBS + 10 ng/mL bFGF) and treatment groups incubated with ectoderm, mesoderm, or endoderm differentiation media. However, the diameter of EBLCs decreased in the three-differentiation media compared with those in the control group (0.001 < *p* ≤ 0.002). Also, the EBLCs in differentiation media possessed a narrow marginal area close to the periphery (black arrowheads) and more central areas (*). Insets show higher magnification. (Scale bar = 150 µm). “A” and “B” denote significant differences and *p* < 0.05 was considered as the significance level.

In Experiment III, after 7 days of culture, the overall morphology of cells and colonies cultured in differentiation media did not differ from those developed in the regular media. However, after 7 days of incubation in differentiation media, EBLCs appeared more translucent and lighter colored compared to day 0 of differentiation (Figure 3). Further, EBLCs developed in differentiation media appeared to have two different, albeit indistinct, areas: a narrow marginal region close to their edges and a more central area. Notably, while the number of gonocytes or EBLCs in differentiation media were not different from that of control groups (0.59 < *p* < 0.93), the average diameter of EBLCs was smaller than that of the control group and was reduced from day 0 to day 7 of differentiation in all tri-lineage differentiation media (0.001 < *p* ≤ 0.002; Figure 3).

### 3.2. Alizarin Red S Staining and Immunofluorescence Assay

#### 3.2.1. Osteogenic Differentiation

All colonies developed in the differentiation group produced hydroxyapatite deposits, while none of the colonies in control group were positive for Alizarin Red S staining. No differences in the extent of differentiation were observed between colonies in the differentiation group. In the control group, gonocytes appeared translucent without color staining and colonies showed a homogenous distribution of dark color. Conversely, all colonies in the differentiation group showed light red staining in the narrow marginal areas and dark red staining in more central areas, indicative of hydroxyapatite deposition (Figure 2E–J). Gonocytes and somatic cells in the differentiation group appeared in light red color following the staining process. The overall distribution pattern of gonocytes, EBLCs, and somatic cells was similar to those observed in the control group.

#### 3.2.2. Pluripotency of Germ Cells and their Differentiation into Derivatives of Three Germinal Layers

**Figure 4:**
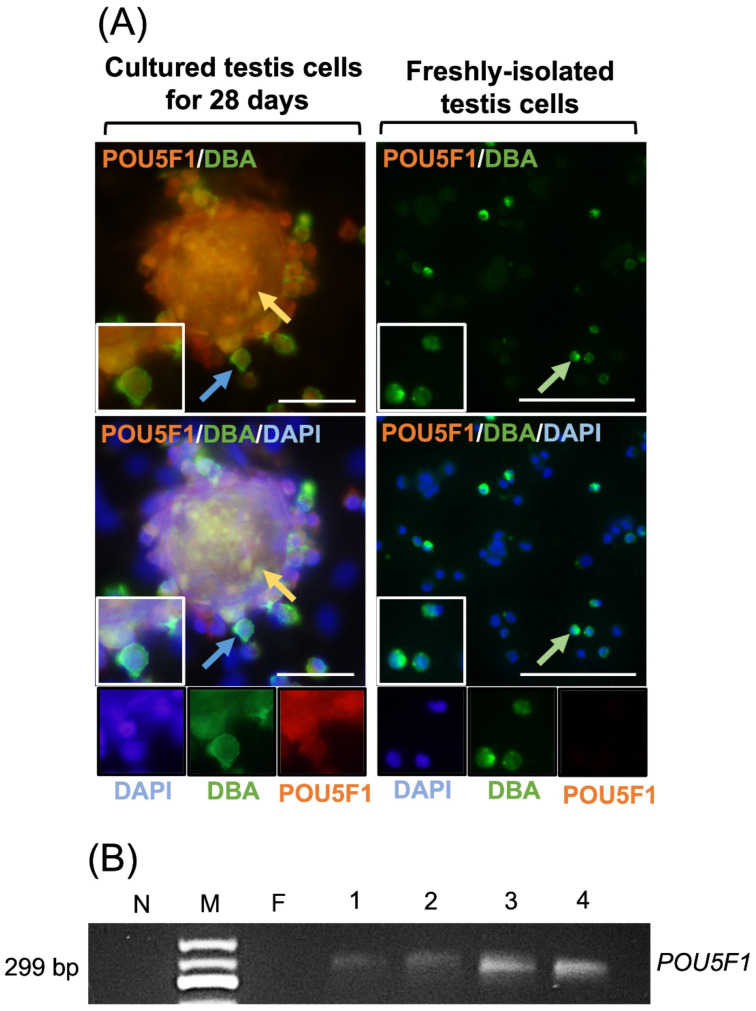
Evaluation of POU5F1 expression as a pluripotency marker and DBA as a gonocyte-specific marker by freshly-isolated and cultured porcine testis cells in regular media. Following isolation of testis cells, gonocytes were positive for DBA (green arrows) but negative for POU5F1. When cultured in regular media, gonocytes (blue arrows) and EBLCs (yellow arrows) were positive for both POU5F1 and DBA (**A**). Testis cells were also negative for POU5F1 immediately after isolation in RT-PCR analyses, while they were positive in samples of cultured cells on days 7, 14, 21, and 28 (**B**). These findings are indicative of spontaneous reversion of MGSCs into more primitive developmental stages. N, no template control; M, 50 bp DNA ladder; F, freshly-isolated testis cell sample; Lines 1–4, cultured cells and colonies in regular media on days 7, 14, 21, or 28, respectively. Insets display higher magnifications (Scale bar = 50 µm).

**Figure 5:**
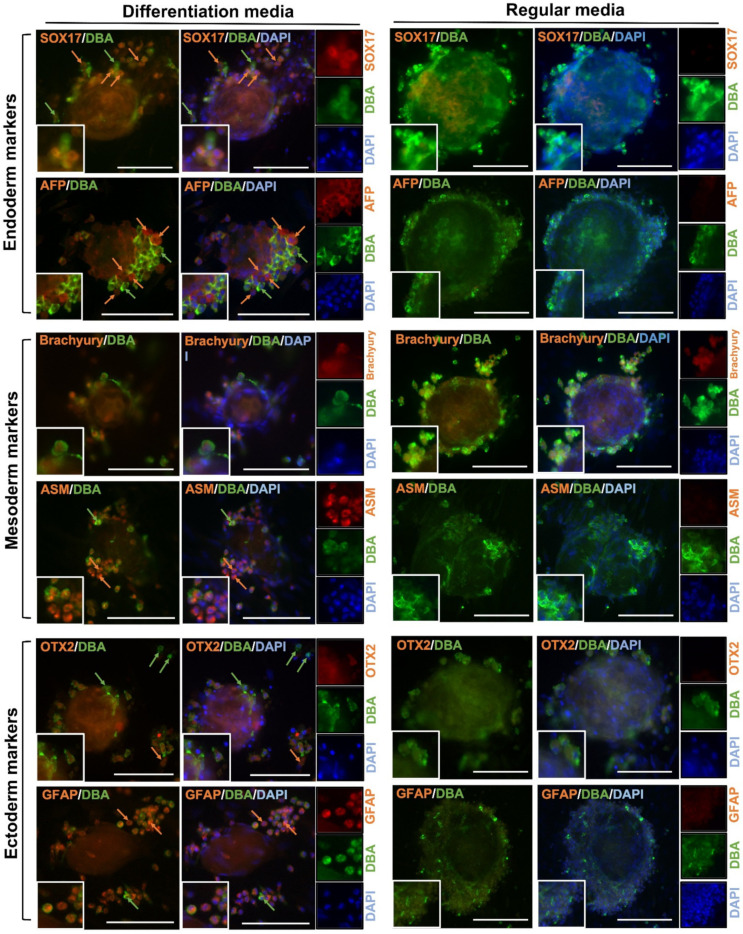
Expression of a gonocyte-specific marker (DBA) and markers of endoderm (SOX17 and AFP), mesoderm (Brachyury and ASM), and ectoderm (OTX2 and GFAP) by cultured gonocytes and their colonies in regular and differentiation media. Overall, gonocytes and embryoid body-like colonies (EBLCs) were positive for DBA. Many gonocytes showed strong expression of DBA (green arrows) but weak expression of lineage-specific markers. Other gonocytes showed strong expression of the lineage-specific markers (orange arrows) when cultured in corresponding media. EBLCs appeared positive for all three lineage differentiation markers. When cultured in regular media, gonocytes, and EBLCs were positive for DBA and Brachyury, but were negative for OTX2, SOX17, AFP, ASM, and GFAP. This may indicate that gonocytes in differentiation media are gradually converting into a new population of cells with similar biomolecular properties as cells of three germinal layer derivatives. Insets show higher magnification (Scale bar = 100 µm).

Freshly-isolated testis cells were negative for POU5F1; however, both gonocytes and EBLCs in regular media appeared positive for POU5F1 on day 28 of culture. Co-localization of DBA and POU5F1 was observed in all germ cells. DBA^+^/POU5F1^+^ cells comprised 42 ± 4% of total number of cells in the regular media. Also, gonocytes and EBLCs in regular media were positive for Brachyury but negative for all other lineage-specific markers. In both regular and differentiation media, germ cells appeared positive for DBA. In differentiation groups, some cells and colonies showed strong expression of DBA but weak expression of designated lineage-specific markers. Conversely, a fraction of cells appeared strongly positive for the designated differentiation marker but showed weak expression of DBA. A similar expression pattern for each lineage-specific marker was observed in the corresponding differentiation media (Figure 4A and Figure 5).

### 3.3. Gene Expression Analyses

**Figure 6:**
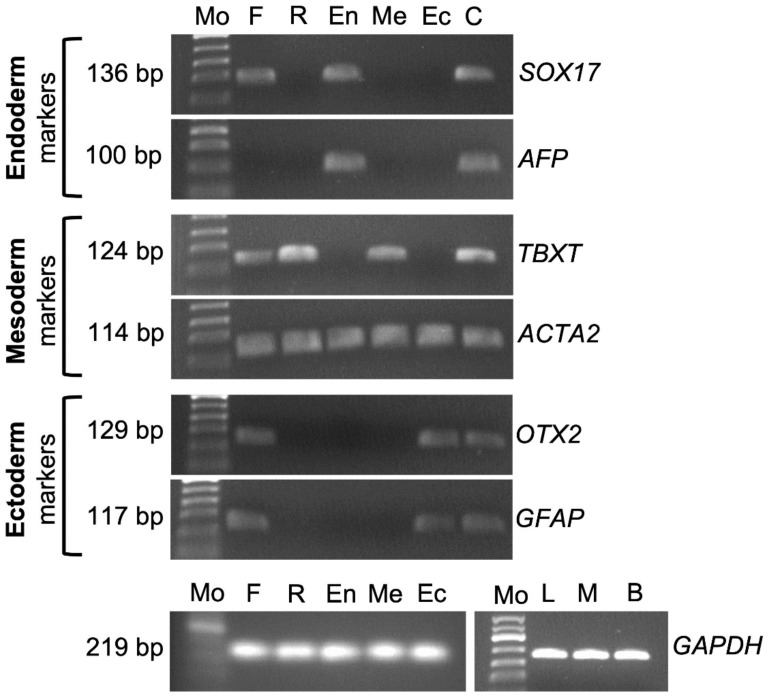
Gene expression analyses of germinal layer-specific markers using RT-PCR. Expression of germinal layer markers was positive in respective differentiation media. Also, freshly isolated testis cell samples were positive for the expression of *SOX17, TBXT, ACTA2, OTX2*, and *GFAP*. Cultured testis cells in regular media (DMEM + 15% FBS) were positive for mesoderm markers (*TBXT* and *ASM*). *ACTA2* expression was also positive in cultures of endoderm and ectoderm differentiation media in addition to cultures treated with mesoderm differentiation media. Mo, 50 bp DNA ladder; F, freshly isolated testis cell sample; R, cultured cells and colonies in regular media; En, cultured cells and colonies in endoderm differentiation media; Me, cultured cells and colonies in mesoderm differentiation media; Ec, cultured cells and colonies in ectoderm differentiation media; C, positive control (liver sample for *SOX17* and *AFP*; muscle sample for *TBXT* and *ACTA2*; brain sample for *OTX2* and *GFAP*); L, liver sample; M, muscle sample; B, brain sample.

Freshly-isolated testis cells were negative for expression of *POU5F1*. However, in Experiment III, cultured cells in regular media displayed *POU5F1* expression on day 7 to day 28 (Figure 4B). RT-PCR for cultured cells in ectoderm, mesoderm, and endoderm differentiation media were positive for *OTX2*/*GFAP*, *TBXT*/*ACTA2*, and *SOX17*/*AFP*, respectively. Samples of freshly isolated testis cells were also positive for *SOX17*, *TBXT*, *ACTA2*, *OTX2*, and *GFAP*. Cells cultured in regular media (DMEM + 15% FBS + 10 ng/mL bFGF) were positive for *TBXT* and *ACTA2*. Also, *ACTA2* expression was evident in samples of endoderm and ectoderm differentiation cultures (Figure 6).

### 3.4. Characterization of Dedifferentiated Cell Implants

#### 3.4.1. H&E Staining

**Figure 7:**
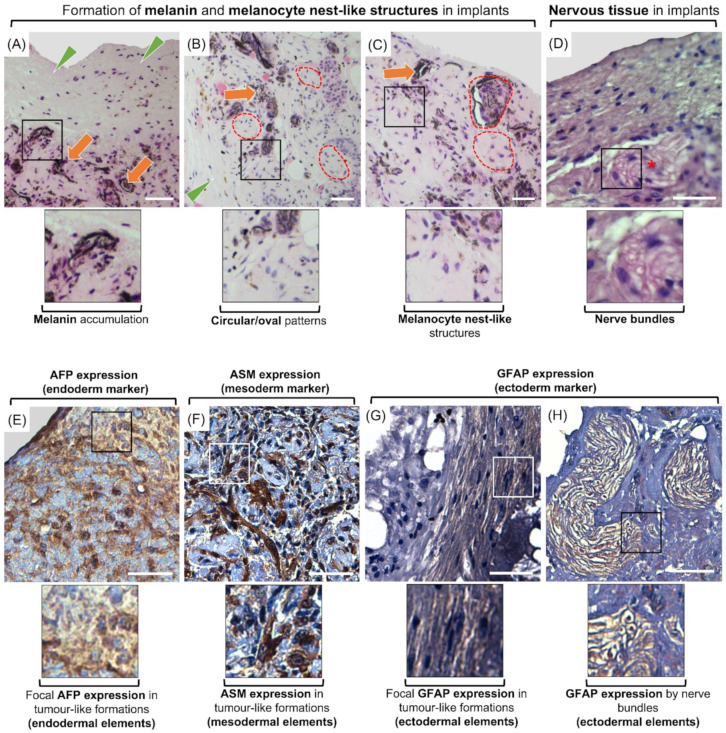
Representative photomicrographs captured from (immuno)histological sections of dedifferentiated cell implants. Implants were stained using H&E (**A**–**D**) or labelled with lineage-specific antibodies including AFP (**E**), ASM (**F**), or GFAP (**G**,**H**). The implants were surrounded by a dense fibrous capsule (green arrowhead) (**A**,**B**). Implants developed into tumor-like growth formations containing circular and/or oval patterns as well as large numbers of melanocytes with remarkable accumulation of melanin pigments (orange arrows) (**A**–**C**). The distribution pattern of the cells resembled nests of melanocytes (red broken lines) (**B**,**C**). Within these formations were also located the bundles resembling those of peripheral nerves (asterisk) (**D**). AFP as endoderm marker was expressed in foci of implanted cells/EBLCs (**E**). Focal AFP expression can be attributed to endodermal elements in tumor-like formations. Also, limited expression of ASM was observed by the cells inside and surrounding the circular patterns (**F**), which can be deemed as the presence of differentiated myofibroblasts. Small foci within the formations and nerve bundle-like structures both appeared positive for GFAP as neuroectodermal marker (**G**,**H**), which can point at development of ectodermal component within the formations. Squares display higher magnification (Scale bar = 50 µm).

Histological examination of the injected cells/colonies (*n* = 16 injected aggregates) revealed gradual formation of cellular structures, which morphologically did not belong to the normal adjacent peripheral tissues. These structures were located at the expected implantation sites, including immediately ventral to the dermis and dorsal to the hypodermis. The morphologies, dimensions and extension of these tumor-like growth formations varied between injection sites and animals. These formations appeared as multicellular dense structures, were present in all implantation sites, and were encapsulated within a dense connective tissue. In sparse areas within the formations, the distribution of cells formed circular and oval patterns. In certain other areas, the formations contained distinguishable groups of cells that resembled melanocyte nests. Furthermore, increased numbers of melanocytes with melanin deposition were detected in cross-sections of all implants, representing the formation of melanomas. In addition, the formations contained bundles resembling those of peripheral nerves (Figure 7A–D). 

#### 3.4.2. Immunohistochemistry

To confirm the differentiation of implanted cells and colonies we used antibodies associated with each germinal layer. Expression of each maker was observed in all samples but were localized to specific areas of the formations. Cells in adjacent foci to the adipose tissue appeared positive for anti-pig-AFP, which was expressed in both the nucleus and cytoplasm. Cells within and surrounding the aforementioned circular/oval patterns expressed ASM. Also, GFAP was expressed in small foci, and within the bundles resembling peripheral nerves. GFAP expression also appeared to be both nuclear and cytoplasmic (Figure 7E–H). 

## 4. Discussion

In previous works as well as in the present study, our laboratory has shown that gonocytes from neonatal porcine testis can spontaneously dedifferentiate and express pluripotency-associated markers when cultured under basic conditions [14,17]. Given the potential of these cells in conversion into a pluripotent state, here we first developed a population of dedifferentiated gonocytes using our previously established culture conditions, evaluated their potential to generate derivatives of three germinal layers in vitro, and assessed their pluripotency and plasticity using an in vivo implantation assay. As such, the present study is the first to show that, upon providing the appropriate media and supplements, dedifferentiated porcine gonocytes and their colonies can undergo osteogenic differentiation in vitro and can convert into cells of the three germinal layers (i.e., ectoderm, mesoderm, or endoderm). Additionally, we showed that implantation of cultured gonocytes and their colonies into the subcutaneous tissue of recipient mice can lead to development of tumor-like growth formations. These formations contain areas which morphologically resembled ectoderm, mesoderm, or endoderm-derived tissues and express markers that are present in all three germinal layers. This observation further confirms the unique potential of gonocytes to spontaneously transform into a pluripotent state under simple in vitro conditions. The dedifferentiation and trans-differentiation potential of germ cells is a remarkable finding that sheds light on their unique conversion potential and can open new avenues of research into their applications in stem cell therapy and regenerative medicine. 

In Experiment I of the present study, we successfully induced osteogenic differentiation in gonocyte colonies. Osteoblasts have mesodermal origin and are specialized mesenchymal cells that synthesize bone matrix [37,38]. Here, we used simple culture conditions to develop pluripotent germline stem cells in a short-term culture (~2 wk) and transformed them into osteogenic cell derivatives with bone matrix deposition potential. Following differentiation, morphological features of EBLCs stained with Alizarin Red S resembled those of in vitro developed osteoblast colonies which typically possess darker color in the central areas and lighter color in the marginal areas [39,40]. This morphological pattern of EBLCs features a dome shape appearance and greater calcium deposition in the central areas compared to the narrow marginal areas. Previously, embryonic chicken SSCs were shown to undergo directional differentiation into osteoblasts by cultivation for multiple passages on a feeder layer and in media composed of multiple growth factors and/or other supplements [41]. Although successful, trans-differentiation rate of chicken SSCs was 75–80%. In contrast, we showed that all EBLCs developed from porcine testis cells transformed into calcium phosphate deposition sites and appeared positive for Alizarin Red S staining; all in a relatively short period of time and without supplementation of multiple exogenous factors. In another study, bovine germ cells were co-cultured with Sertoli cells for 21 days in a differentiation induction media to become trans-differentiated into osteoblasts followed by confirmation of differentiation using Alizarin Red S staining [42]. Although multiple studies have already shown mesenchymal and ES cell potential to convert into osteogenic cell lineages [43,44], successful generation of osteogenic progenitors from porcine germ cells acts as an even stronger indication of similar potential in human germ cells, given the anatomical and physiological similarities between pigs and humans. As such, our findings introduce a new avenue for application of these cells in bone regeneration and matrix formation. Of notable importance, using MGSCs for the purpose of bone regeneration in an autologous manner does not carry the same ethical concerns associated with the use of ES cells, nor the biosafety risks associated with the use of iPS cells. 

In addition to the osteogenic differentiation, we also examined the conversion potential of cultured gonocytes into cell derivatives of all three germinal layers. As mentioned above, Experiment II was not continued due to limited cell growth and negative expression of lineage-specific markers following brief exposure to the differentiation media. Limited cell growth in Experiment II can be attributed to an underdeveloped somatic cell monolayer, which is essential for survival of non-adherent gonocytes [14,26]. We suggest that perhaps the frequent replenishment of the differentiation media in Experiment II led to physical detachment of the monolayer and/or removed essential components that were naturally produced by cultured cells. However, in the immunofluorescence results of Experiment III we showed decreased intensity of DBA expression and increased intensity of lineage-specific markers, including AFP/SOX17 in endoderm differentiation group, ASM in mesoderm differentiation group, and GFAP/OTX2 in ectoderm differentiation group, all indicative of gradual conversion of gonocytes into other cell lineages.

The expression of transcription factors examined in the present study are critical for normal formation and development of germinal layers during embryonic development [45,46,47,48,49,50,51]. In the present work, we used differentiation kits designed for in vitro differentiation of human ES cells into three germinal layers. For each germinal layer, the manufacturer has provided one of the main and validated biomarkers for confirmation of differentiation into endoderm, mesoderm, or ectoderm. To increase the reliability of our observations and further confirm successful trans-differentiation, we evaluated the expression of a secondary set of markers that have been previously used and validated for each germinal layer [10,52,53,54,55,56,57,58]. Here, positive expression of SOX17 in freshly isolated testis cells is in agreement with previous studies on isolated human PGCs and gonocytes [59,60]. Conversely, negative expression of SOX17 in cultures of regular media may be indicative of germ cell transformation into more primitive developmental stages. SOX17 is a key transcription factor in the development of endoderm as its mutation leads to defective intercellular transport in endoderm [60,61]. Additionally, we used AFP as a secondary endoderm marker. Expression of AFP is typically upregulated in nonseminomatous germ cell tumors [62] and expectedly was not expressed in our fresh testis cell samples. Here, mesoderm and ectoderm cultures were also negative for AFP, perhaps due to the lack of chemical components required for directing cell differentiation towards definitive endodermal lineages. Components of endoderm differentiation media such as bFGF, Wnt-a, and Activin A play important roles in enhancement of definitive endoderm-associated gene expression and cell viability in a dose dependent manner [63,64,65,66]. The same interplay can be speculated between these components in our culture system, leading to expression of endodermal gene transcripts. 

Here, we also showed mesoderm differentiation cultures became Brachyury^+^ while endoderm and ectoderm differentiation cultures appeared Brachyury^−^. Brachyury is a factor required for posterior mesoderm formation [67]. In agreement with our observations, a previous study on mouse germ cells showed fresh testis lysates and germ cell enriched cultures were positive for Brachyury, which the authors concluded was essential for mouse germ cell self-renewal [68]. Interestingly, multipotent germline stem cells developed from murine testis cells were also positive for Brachyury [69]. This further supports our finding regarding Brachyury^+^ cultures in regular media, which were previously demonstrated to express pluripotency-associated markers [14,17]. Additionally, ASM as a secondary marker for mesoderm differentiation was positive in all samples. This could have occurred due to contaminating peritubular myoid cells and/or blood endothelial cells; however, in immunocytochemistry, ASM expression was localized to the cells surrounding the EBLCs, which also weakly expressed DBA. This may point at gradual transformation of DBA^+^ cells to cells with mesodermal origin. Identification and positive expression of ASM has been also reported in previous studies on primate testis cell culture [70,71]. ASM is one of the actin isoforms responsible for mechanical tension of the cells [72]. 

Here, similar to SOX17, the expression of OTX2 and GFAP was downregulated in germ cells during culture in regular media. OTX2 is required for neuroectoderm differentiation [23,73,74,75] and is inherently expressed in porcine brain and reproductive tissues [76,77]. Additionally, Leydig cells possess neuroendocrine features and are immunopositive for neuroectodermal and astrocyte markers such as GFAP [78,79]. Thus, positive GFAP expression by freshly isolated cells can be explained by the presence of Leydig cells, which once isolated and cultured in regular media, perhaps downregulated its expression. 

In Experiments I and III, migrating gonocytes formed clusters and colonies which transformed into EBLCs. Although smaller in size, the morphology of the EBLCs in Experiment III was comparable to those of Experiment I. The presence of two indistinct peripheral and central areas in EBLCs can be explained by their dome-shape appearance, which normally have thinner marginal areas and thicker central areas. This can lead to a more translucent edge and opaque center in EBLCs.

We have previously shown that MGSCs in regular media dedifferentiate and express a number of ES cell markers [14]. Similarly, to confirm dedifferentiation of cultured MGSCs in regular media into primitive developmental stages, we examined their expression of POU5F1 as one of the important and widely used ES markers [30,31,32]. As demonstrated previously, unlike freshly-isolated testis cells which are negative for this marker, cultured cells are positive POU5F1 expression [14,17]. Here we showed that all MGSCs in culture conditions appeared both DBA- and POU5F1- positive. This further confirms that MGSCs are able to spontaneously revert to a dedifferentiated state and express markers that are mutually present in ES cells. As such, in the present study we also used an implantation assay to evaluate their tumorigenic potential and examined the expression of selected germinal layer biomarkers (i.e., AFP, ASM, and GFAP) in retrieved samples. In all injection sites, multicellular tumor-like formations contained foci with positive cells for AFP, ASM, or GFAP. AFP is a known marker for nonseminomatous germ cell tumors [80], and its expression may point at development of endodermal component within our observed formations [81,82,83]. ASM expression was similarly observed in sparse locations of our retrieved samples. ASM expression is associated with mesodermal differentiation in tumors derived from pluripotent stem cells [84,85]. Here, we also observed GFAP expression in focal areas within the formations. The morphology and pattern of diffuse GFAP-positive areas partly resembled those observed in previously reported glial cell neoplastic tissues containing polygonal and spindle shape cells with indistinct cellular borders [86]. Additionally, nerve bundle-like structures within these formations appeared positive for GFAP, which may suggest the presence of a neuroectodermal component. Increased GFAP expression has been attributed to neuroectodermal component in teratomas [87,88]. Importantly, excessive accumulation of melanin and development of melanocyte nests further confirms the presence of ectodermal tissue in the developed tumor-like formations. Thus, the overall morphological and immunohistochemical attributes of the implanted cells and EBLCs point at their potential contribution to formation of tumor-like structures which contain endodermal, mesodermal, and ectodermal components. The tumorigenic potential is one of the staple characteristics of ES- or iPS cells derived from somatic cells [85]. Additionally, by definition, pluripotent stem cells can give rise to cells of all three germinal layers, whereas multipotent stem cells can differentiate to particular tissue types/cell lineages but not all [89]. Given that, similar to ES- and iPS cells, our developed germline-derived stem cells have the ability to differentiate into cells of all three germinal layers, generate teratomas, and express pluripotency markers, they can be classified as pluripotent stem cells. As such, gonocytes in our primary culture conditions spontaneously underwent reprogramming into a pluripotent state in a relatively shorter time (~4 wk post-implantation) as confirmed by the implantation assay. In addition, we successfully converted these cells into cell derivatives from all three germinal layers, which has not been achieved previously. Harvesting germ cells from primary culture is important since multiple passages and removal of the cells from their microenvironment causes unwanted changes to their physiological and biochemical properties [90]. Such changes can generate confounding factors for their downstream applications and pose negative effects on their proliferation and differentiation potential at genetic or epigenetic levels [90].

Therefore, our results substantiate the conclusion that neonatal germline stem cells present dedifferentiation and trans-differentiation capabilities. As such, the present work sets the stage for future studies using human germ cells for potential use in regenerative medicine with important advantages over the use of ES or iPS cells for similar applications. For instance, we propose that considerations be given to the use of testicular biopsies both as a source for sperm and stem cell production. Using an individual’s endogenous germ cells for development of pluripotent stem cells holds great promise for downstream applications in stem cell therapy of the same individual. Such an autologous application of germline-derived pluripotent stem cells is expected to be far less problematic from the ethical and biosafety aspects. This is especially the case when considering that the testicular biopsies collected prior to the start of cancer treatment from preadolescent patients are routinely cryopreserved and hence can serve dual purposes; not only can they be considered as the source of germline stem cells for potential restoration of fertility, they may also be viewed as a potential source for derivation of pluripotent stem cells for other tissue regeneration applications. Importantly, autologous application of germline-derived stem cells also eliminates or limits issues related to immunorejection and the various biosafety and ethical issues related to the genetic manipulation or acquisition of stem cells from allogeneic, xenogeneic, iPS, or ES sources to derive stem cells for cell-based therapy to replace cells and tissues perished during the course of cytoablative cancer treatments. However, as with any other potential source for stem cell therapy, due diligence should be applied to ensure safety of the germline-derived stem cells, especially as related to the potential presence of tumor cells. 

## 5. Conclusions

Neonatal porcine gonocytes represent a promising source of adult stem cells. In our culture conditions, these stem cells undergo dedifferentiation into more primitive developmental stages. When implanted into recipient mice, the cultured gonocytes and their colonies develop formations with morphological and biomolecular characteristics of all three germinal layers, which further confirms their plasticity. Furthermore, cultured gonocytes and their colonies effectively convert into derivatives of ectodermal, mesodermal, and endodermal germinal layers in trans-differentiation media. In vitro dedifferentiation and subsequent conversion of neonatal gonocytes into osteogenic cells and derivatives of three germinal layers are important observations since this in vitro system can provide an easily accessible and abundant source of adult stem cells for numerous downstream applications in regenerative medicine. More importantly, unique conversion potential of neonatal gonocytes into other cell types makes them a potentially attractive autologous pluripotent cell source since their use eliminates ethical implications and the biosafety risks associated with viral vectors to induce pluripotency, and they can be easily produced in vitro without addition of multiple extrinsic factors. The capacity of neonatal germ cells to differentiate into cells of all embryonic germinal layers highlights their importance and warrants further research including on their putative differentiation triggers. Such studies may put these cells in a privileged position to treat tissues of high interest in regenerative medicine, especially those that present low regeneration rates such as neural tissue, tendon, or cartilage when compared to other better studied multipotent stem cells. More specifically, the testicular biopsies obtained from preadolescent boys can potentially serve as a dual source of germ cells for fertility restoration and germ cell-derived pluripotent stem cells for development of somatic cells and tissues. As such, MGSCs offer an autologous alternative stem cell source to the available adult stem cells such as cord blood stem cells with similar potentials. It goes without saying that any in vivo application of these cells should be preceded with extensive assessments of any potential risk for tumor development or other biosafety risks.

## Figures and Tables

**Table 1:** Antibodies used for immunofluorescence and immunocytochemistry.

Antibody	Supplier	Catalogue No.	Dilution
Rabbit anti-POU5F1	Abcam	AB18976	1:200
Goat anti-OTX2	R&D Systems	SC031B	1:200
Mouse anti-GFAP	Novus Biological	NBP1-05197SS	1:200
Goat anti-Brachyury	R&D Systems	SC030B	1:200
Mouse anti-ASM	Novus Biological	NBP1-33006	1:200
Goat anti-SOX17	R&D Systems	SC019B	1:200
Rabbit anti-AFP	Novus Biological	NBP1-76275	1:200
Alexa Fluor 594 goat anti-rabbit	Abcam	AB150088	1:200
Alexa Fluor 594 goat anti-mouse	Abcam	150116	1:200
Alexa Fluor 594 rabbit anti-goat	Abcam	150148	1:200

**Table 2:** RT-PCR primer sequences for gene expression analyses.

Target Name	Direction	Primer Sequence (5′-3′)	Annealing Temperature (°C)	Product Size (BP)
*POU5F1*	Forward	AGAGAAAGCGGACAAGTA	51.7	299
	Reverse	ATCCTCTCGTTGCGAATA		
*OTX2*	Forward	TTTATCTGGTCTCTCTCCCTCTC	61	129
	Reverse	GTTAGTGGTGGAAAGTGGTAGG		
*GFAP*	Forward	CAGAGCAGGACCGAGTTTATG	61	117
	Reverse	CATAAAGAGAAGAGGGAAGGACAG		
*TBXT*	Forward	GGGATTTGCTTCTGGGTCTAA	63.7	124
	Reverse	GTTGAGAAGTCACTGGACAGAG		
*ACTA2*	Forward	CTGGGTCTGAGTCTTAGCTTTC	63.7	114
	Reverse	GATAGGATGGCTGTGTGGATT		
*SOX17*	Forward	CATCTCAAGTGACCCTAGTCTTTAC	61	136
	Reverse	GTTGAATCTTGAGGTCTGCCT		
*AFP*	Forward	GCTCCATCTCCTTGCTTTCT	66.1	100
	Reverse	AAGAGATGCCCATAAACCCTG		
*GAPDH*	Forward	TCGGAGTGAACGGATTTG	62	219
	Reverse	CCTGGAAGATGGTGATGG		

## Data Availability

The visual and numeric data included in this article to further support the conclusion are available upon request.
